# Orange-derived and dexamethasone-encapsulated extracellular vesicles reduced proteinuria and alleviated pathological lesions in IgA nephropathy by targeting intestinal lymphocytes

**DOI:** 10.3389/fimmu.2022.900963

**Published:** 2022-08-31

**Authors:** Wang Zhang, Ye Yuan, Xiang Li, Jiao Luo, Zhanmei Zhou, Lei Yu, Guobao Wang

**Affiliations:** ^1^ Renal Division, Nanfang Hospital, Southern Medical University, National Clinical Research Center for Kidney Disease, State Key Laboratory of Organ Failure Research, Guangzhou, China; ^2^ Department of Anatomy, School of Basic Medical Sciences, Southern Medical University, Guangzhou, China

**Keywords:** IgA nephropathy (IgAN), intestine immunity, extracellular vesicle (EV), immunosuppressive therapy, Peyer’s patches

## Abstract

Current evidence highlights the critical role of the gut-kidney axis in the pathogenesis of IgA nephropathy (IgAN). However, few attempts have been made to explore targeted intestinal immunity therapy. This research aims to develop an oral intestine targeting medication based on extracellular vesicles (EVs) and investigate its therapeutic efficacy in IgAN. EVs were isolated from orange juice and electroporated with dexamethasone sodium phosphate (DexP). After oral administration, EVs-DexP was picked up by lymphocytes in the submucosal area of ileocecum. EVs-DexP outperformed DexP not only in suppressing lymphocyte stimulation *in vitro* but also in alleviating renal pathological lesions in the IgAN mouse model. Clinical improvement was accompanied by a reducing IgA secreted by the intestine and a decreasing IgA + B220 + lymphocytes in Peyer’s patches. The present study develops a cost-effective, biofriendly EVs-based glucocorticoid strategy for IgAN.

## Introduction

IgA nephropathy (IgAN) is the most common primary glomerulonephritis worldwide, especially in Asia, with heterogeneous clinical and pathological phenotypes. It is estimated that 20–40% of IgAN patients develop ESRD within 20 years from the time of diagnosis (1). The recent IgAN landmark trials of STOP-IgAN (2)and TESTING (3) raised deep concern about the issue of corticosteroids/immunosuppressive therapy-related infection, despite potential benefits of preventing renal progression (4).

The gut-kidney axis in IgA nephropathy has been comprehensively reviewed by Coppo (5), who concluded that genetic background, B cell activity, IgA synthesis, gut-associated lymphoid tissue intestinal immunity(GALT), and diet may all have a role in the development and progression of IgA nephropathy. Peyer’s patches (PPs), the inductive sites producing immunocompetent primed B cells, are supposed to be a key element in IgA production. Nevertheless, limited attempts have been performed to explore intestinal immunity targeted therapy in IgAN.

The past decade has witnessed dramatic progress in nanotechnology combined with modern medicine. Extracellular vesicles (EVs), characterized by nanoscale sizes ranging from 30 to 200 nm and a (phospho) lipid bilayer structure, have recently gained much interest as promising candidates for drug delivery. They are nanoparticles derived from a variety of cells and found in almost all biological fluids (6), mediating physiological and pathological processes. However, heterogeneous origination and composition, inconsistent stability in circulation and metabolism, and low quantities yield by mammalian cells (7) challenged their role as drug carriers in clinical application. Plant-derived nanoparticles emerged as feasible natural drug carriers considering a stable physicochemical property, biosafety, and large-scale production (8).

Herein, we set out to isolate EVs from oranges and evaluate the capacity of this plant-derived nanoparticle as an oral drug delivery vehicle. Our findings that EVs were taken up by ileocecal lymphocytes after oral administration further inspired us to load dexamethasone sodium phosphate (DexP), one of the highly potent glucocorticoids, into EVs and investigate its immunosuppressive effect *in vitro* and therapeutic efficacy in the IgAN mouse model. Our findings would provide a novel bio-friendly approach to treat IgAN *via* regulating intestinal immunity.

## Materials and methods

### EVs isolation

Orange juice was squeezed and filtered by gravity through a Whatman filter paper. Collect the filtrate 125mL and dilute the sample using protease inhibitor and PBS to a final volume of 250mL. The protease inhibitor cocktail, a mix of 1.67mL 1M sodium azide, 2.5mL of 100mM PMSF, and 0.5mL of 1mM leupeptin stock solutions, was immediately added as recommended by Christopher, etc. (9) Then low-velocity centrifugation was performed at room temperature: centrifuge at 4,000 × g for 20 min to remove cells and large debris, then at 8,000 × g for 20 min, followed by 15,000 × g for 20 min to remove cellular debris. Filter the supernatant by 0.45μm syringe filter. The next step was high-velocity ultracentrifugation at 4°C: centrifuge the supernatant in polycarbonate ultracentrifuge tube at 170,000 × g for 90 min to pellet the crude extracellular vesicles fraction. Carefully discard the supernatant and resuspend the pellet in a small volume of PBS. Then vortex it rigorously for at least 10 min to break all aggregation and obtain a suspension of primary nanovesicles.

### Quantification

Particle number was measured by nanoparticle tracking analysis (NTA) using ZetaView Particle Metrix. EVs were lyophilized and weighed up, and total proteins were measured using BCA assay.

### Particle size and surface charge

Collected samples were diluted to avoid inter-particle interaction. Size distribution and zeta potential were assessed by Zetasizer Nano ZS (Malvern Instruments, UK).

### Transmission electron microscopy

EVs pellets were resuspended in PBS and spotted onto a carbon-coated copper grid. The excess liquid was removed, and filter paper was used to drain the grid; a drop was negatively stained with 3% phosphotungstic acid and loaded onto the grid for 5 minutes. The grid was then dried at room temperature. Finally, the samples were observed under a HITACHI H-7650 transmission electron microscope operated at 80kV.

### Lipidomic analysis

EVs samples in chloroform/methanol/water (1/1/1, v/v/v) solution were vortexed for 30 mins and centrifuged at 1,500 × g for 10 mins. Organic phage was collected and transferred to a new tube and lyophilized using nitrogen. Dried metabolites were reconstituted in 400μl isopropanol/methanol (1/1, v/v) solution, vortexed, centrifuged at 23,500 × g for 10 min at 4°C, and the supernatant was analyzed using liquid chromatograph-mass spectrometry (LC-MS) (Thermo, Ultimate 3000LC, Orbitrap Elite). A Kinetex C18 column (100 × 2.1mm, 1.9μm) and the following gradient: 0-2 min 30% mobile phase B; 2-2.1min 55% B; 2.1-12 min 65% B;12-18 min 85% B; 18-25 min100% B; 25-30 min 30% B, was applied for the experiment. Mobile phase A was acetonitrile/water (3:2, v/v), 10mM ammonium formate and 0.1% formic acid. Mobile phase B was acetonitrile/isopropanol (1:9, v/v), 10mM ammonium formate and 0.1% formic acid. The flow rate was 0.3 ml/min, and the column was at 45°C. The MS data files were processed using Thermo package software Lipid Search for producing a list of lipid names and peak areas.

### Drug encapsulation

A mixture of EVs and dexamethasone sodium phosphate (DexP) in different proportions were gently blended in 0.5ml of electroporation buffer (125mM NaCl, 5mM KCl, 1.5mM CaCl2, 10mM glucose, 20mM HEPES, pH 7.4) at 4°C. After electroporation at 300 V for 15ms in 0.2cm electroporation cuvettes by a Gene Pulser II Electroporator (Bio-Rad, USA), the mixture was incubated at 37°C for 30 min to ensure the plasma membrane of the EVs fully recovered. Residual DexP and electroporation buffer were removed by Milli-Q water using a regenerated cellulose dialysis membrane (Spectra/Por 4 Dialysis Membranes, Carl Roth) with 6-8kDa molecular weight cut-off (MWCO). Change the Milli-Q water every 4 hours, and the duration of dialysis is 24 hours. The retained EVs were incubated with 1% Triton-X 100 (SigmaAldrich, Schnelldorf, Germany)9 for 4 hours on ice for lysing. Then the lysate was subjected to intermittent sonication for 10 minutes to ensure the (phospho) lipid bilayer of EVs was fully ruptured. The amount of encapsulated DexP was measured by detecting the absorbance at 242nm using UV-spectrophotometry. Entrapment Efficiency(%EE) = (total amount of DexP added - unencapsulated DexP “)/total amount of DexP added] *100%.

### Toxicity

For cytotoxicity assays, cells (3×10^5^/well) were cultivated on 96-well plates, then added with the indicated concentration of drugs in 10% FBS RPMI 1640 containing 5 ug/ml ConA and 3U/ml IL-2. Cell proliferation was detected after 24-hour incubation with cell counting kit-8 (DOJINDO Laboratories, CK04) at 450 nm. *In vivo*, wild-type female BALB/c mice (5- or 6-week-old) were treated daily with EVs (80 mg/kg b. wt.) for two weeks. Serum levels of alanine aminotransferase (ALT) creatinine (Cr) and urea nitrogen (BUN) were analyzed by the AU480 Chemistry System (Beckman Coulter System).

### Stability

The stability of EVs was examined in acidic solution (0.01M hydrochloric acid, PH=2), alkaline solution (0.1M sodium bicarbonate, PH=8), and artificial gastric and intestinal fluid respectively. The artificial gastric fluid solution was prepared according to Polish Pharmacopoeia IX by the dissolution of 2.0g NaCl and 3.2g pepsin in quadruple-distilled water. Then, 80 mL of 1M hydrochloric acid was added to adjust the pH, then supplemented with quadruple-distilled water to 1000mL. Artificial intestinal juice was prepared by dissolving 6.8g of potassium dihydrogen phosphate into 500mL of distilled water, using 0.1 M NaHCO3 solution to adjust the PH to 6.8. Then 10g of pancreatin was mixed, and distilled water was supplemented to 1000mL.

### *In vivo* distribution of EVs

Orange-derived EVs were labeled with near-infrared fluorescent dye Dil (20 μM) by incubation at 37°C for 30 min, followed by centrifugation at 10,000×g for 30 min to remove unbound dye. Mice that had been fasted for 24 hours were gastrically given 200ul Dil-labeled EVs (80 mg/kg EVs protein) and were euthanized at 0h, 2h, 4h, and 6h following gavage. Imaging *ex vivo* was performed on the stomach, gut, spleen, liver, kidney, and bladder. Fluorescence intensities were assessed using Carestream MS FX Pro *In Vivo* Imaging System with customized wavelength filters for excised organs. The relative intensities were measured and compared with an equal amount of unlabeled EVs treated control.

### Isolation of Peyer’s patches lymphocytes

Intestinal Peyer’s patch lymphocytes were collected as described previously (10). Briefly, euthanize the mouse and aseptically remove the small intestine from the duodenum to the cecum. PPs are excised from the intestines and gently forced through a steel mesh grid. Gravity sediment the resulting cell suspension through 4°C CMF/HEPES for 10 minutes, discard the debris pellet and collect the supernatant. Pellet cells by centrifuging 20 min at 850 × g, in a swinging-bucket rotor, 4°C. Resuspend pellet and subject to Percoll fractionation. Lymphocytes will be recovered from a distinct band formed at the interface of the 100% and 40% Percoll.

### Flow cytometric characterization of Peyer’s patch lymphocytes

Murine PPs lymphocytes were isolated and cultured for 24h in 12-well plates with a cell count of 0.5-1×10^7^ in 3ml cell suspension per well. For viability assay, lymphocytes were stimulated with 0.5ug/ml ConA and 3U/ml IL-2 in each well for 24h. The inhibitory effect of EVs (0.5ug/well), DexP (0.1ug/ml), and EVs-DexP (0.6ug EVs electroporated with 0.3ug DexP) were then determined. The following antibodies were used for FACS analysis: Fixable Viability Stain 780(BD Horizon™, 565388); PE Hamster Anti-Mouse CD3(BD Pharmingen™, 553063); FITC Rat Anti-Mouse CD4 (BD Pharmingen™, 553729); APC Hamster Anti-Mouse CD69(BD Pharmingen™, 560689); FITC Rat Anti-Mouse IgA (Invitrogen, 11-4204-82); PE Rat Anti-Mouse CD45R/B220(BD Pharmingen™, 553089). Cells were analyzed using a FACScan (Becton Dickinson, San Jose, California, USA).

### The IgAN mouse model and treatments

Male Balb/c mice, 6- or 8-week-old (weight 25 ± 5g), were obtained from the Laboratory Animal Center (Southern Medical University, China). The routine urine test was checked after one week of pre-feeding. Balb/c mice were fed standard food and had free access to distilled water. On the 13th week, all Balb/c mice were killed.

Twenty-four mice were randomized into four groups:

Control group (n =6): mice were given oral acidified water on alternative days until death. After 6 weeks and on the 9th week, they were injected *via* tail veins with saline with the same quantity and time as the model group.

IgAN model group (n =6): mice were orally given 0.1% bovine serum albumin (BSA) (Sigma Chemical Co., St. Louis, MO, USA) with acidified water (6 mM HCl), 0.4mL for each, on alternate days. After 6 weeks, each mouse was injected *via* tail veins with 0.1 ml 1% BSA buffer solution at a fixed time, once a day, for 3 days. From the 9th week on, the mice were injected with staphylococcal enterotoxin B (SEB) (the Academy of Military Medical Sciences, Beijing, China), diluted by sterile saline with 0.4 mg/kg, once a week, for 3 weeks.

DexP treatment group (n =6): from the 6th week on, each IgAN mouse was intragastrical given DexP 1.6mg/kg every other day until the end of the 12th week.

EVs-DexP treatment group (n =6): from the 6th week on, each IgAN mouse was intragastrical given EVs-DexP (100ug EVs and 40ug DexP were electroporated) every other day until the end of the 12th week.

Spot-urine sample was collected, and urinary albumin/creatinine ratio (ACR) was evaluated before and after treatment using a QuantiChrom™ Protein Creatinine Ratio Assay Kit (DPCR-100, Bioassay Systems, Hayward, CA, USA). Serum creatinine and aminotransferase levels were measured with commercial kits according to the manufacturer’s instructions. Kidney tissues were fixed with 10% paraformaldehyde and embedded in paraffin. They were cut into 3μm tissue sections and stained with PAS methods. The stained renal tissue sections were examined and scored by pathologists under an optical microscope.

All mouse care and experiments were approved by the Institutional Animal Care and Use Committee (IACUC) of Nanfang Hospital. All experimental procedures and animal care were carried out under the guidance of the Ethics Committee to minimize the suffering of animals.

### Immunohistochemistry and immunofluorescence staining

For IHC staining, formalin-fixed and paraffin-embedded small intestine sections were incubated with primary antibodies against IgA (Abcam, ab97234) and analyzed using streptavidin peroxidase detection system (Maixin) according to the manufacturer’s protocol. DAB (Maixin) was used as an HRP-specific substrate. Staining intensity for IgA was ranked using a 5-point scale from 0 (unstained) to 4 (very intensively stained). The histopathological evaluations were performed by three independent pathologists. Every group and tissue examination were blinded.

The kidney and small intestine tissue for IF staining was cut into frozen sections and fixed with acetone for 1 min. After fixation, they were blocked with 2% bovine serum albumin diluted by PBS at room temperature for 1 h. They were washed with PBS three times and incubated with FITC-labelled goat anti-mouse IgA (Abcam, ab97234) at 37°C for 40min. After washing with PBS three times, they were mounted with anti-quenching tablets and observed under a confocal microscope. Cell nuclei were stained with DAPI. Images were quantified by counting the number of positive nuclei and divided by the total number of nuclei.

### Statistical analysis

All data were analyzed by the SPSS18.0 statistical software. A two-tailed, unpaired, or paired Student t-test was used to compare the variables of two groups, and one-way or two-way ANOVA was performed for multi-group comparisons. With homogeneity of variance between two groups, the LSD test was used. With the heterogeneity of variance between groups, the Games-Howell test was employed. Co-localization of IF and the analysis of mean of interest of domain (IOD) in IHC staining were calculated with Image-Pro Plus. P <0.05was noted statistically different. Statistical details are included in the respective figure legends.

## Results

### Preparation and characterization of DexP-packaging EVs

**Figure 1:**
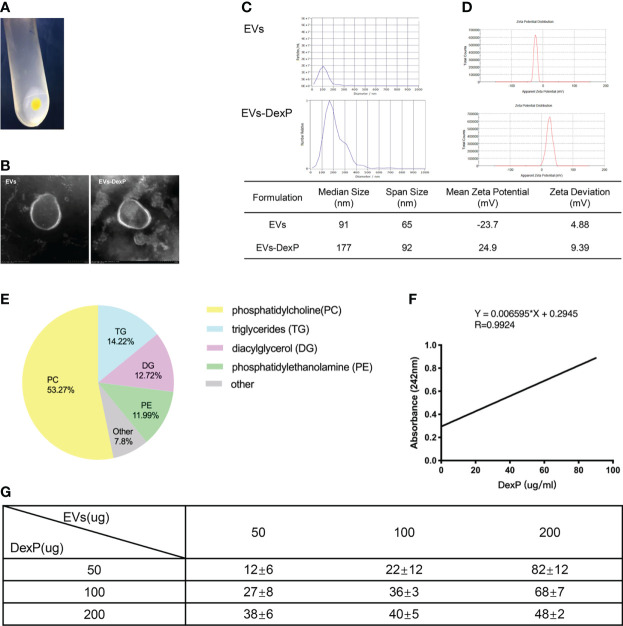
Characterization of EVs and EVs-DexP. **(A)** EVs pellets extracted from orange juice by ultra high-speed centrifugation. **(B)** Electron microscopic image of EVs and EVs-DexP. **(C)** Size distribution of EVs and EVs-DexP. **(D)** Zeta potential of EVs and EVs-DexP. **(E)** LC-MS-based lipid profiling of orange-derived EVs. **(F)** The standard curve was determined by varying concentrations of DexP and its corresponding absorbance at 242 nm using a UV-spectrophotometer. **(G)** Entrapment Efficiency (EE) at different concentrations of DexP and EVs *via* electroporation (time constant=10 ms and voltage=300V). EE (%) = (total amount of DexP added - unencapsulated DexP)/total amount of DexP added *100%.

Extracellular vesicles were isolated from orange juice using the ultra-high-speed centrifugation method (**Figure 1A**). Negatively stained cup-shaped membrane nanovesicles of 100 to 150 nm were discovered using electron microscopy (**Figure 1B**). By BCA quantification, we obtained 5.65 ± 0.51mg of total protein in 758.4 ± 30.3mg pellets from 100ml juice. There were 1.1×10^11^ particles in 100μg/ml EVs suspension, and the median size of EVs was 91nm according to NTA (**Figure 1C**). Zeta potential ranging -23.7 ± 4.88 were examined by dynamic light scattering (DLS) (**Figure 1D**). Orange-derived EVs had 53.27% phosphatidylcholine and comparatively low triglycerides (14.2%), diacylglycerol (12.7%), and phosphatidylethanolamine (12.0%), according to LC-MS lipid profiling (**Figure 1E**).

Electroporation is a process in which a hydrophilic pore is formed in the cellular membrane in a condition of an external electric field, allowing chemicals, DNA, RNA, or drugs to flow through. Membrane integrity quickly recovers after drug loading into the vesicle interior through transient holes. Because dexamethasone is lipid-soluble and EVs are made up of a (phospho) lipid bilayer, it’s challenging to encapsulate Dex intravesicular rather than sandwiched between double-membrane structures or residing at their bilayer surface. Thus, we chose its water-soluble pro-drug dexamethasone phosphate (DexP) to be encapsulated, which is known to be processed in phagocyte lysosomes to deliver active dexamethasone into the cell cytoplasm. After drug loading, EVs still maintained the integrity of the double membrane structure (**Figure 1B**), with the size increased from 91 to 177nm (**Figure 1C**) and the mean zeta potential changed to 24.9 mV (**Figure 1D**). We investigated the encapsulation efficiency (EE) at various concentrations of EVs and DexP in a 200ul electroporation buffer system with a time constant of 10ms and a voltage of 300V. We firstly constructed the standard curve of absorbance values for different DexP concentrations in electroporation buffer (**Figure 1F**), and ecapulated DexP content was calculated accrording to the detected absorbance at 242nm using UV-spectrophotometry after the EVs membrane was ruptured. When 50ug or 100ug DexP is introduced, EE raises as the amount of EVs increases from 50ug to 200ug. When the amount of DexP was larger than or equal to the amount of EVs at 200ug, however, the proportional increase was reduced. When the EVs: DexP concentration ratio was 1:2 or even 1:4, the optimal encapsulation efficiency was calculated to be 70-80% (**Figure 1G**).

### Stability, safety, and distribution of orally administered EVs

**Figure 2:**
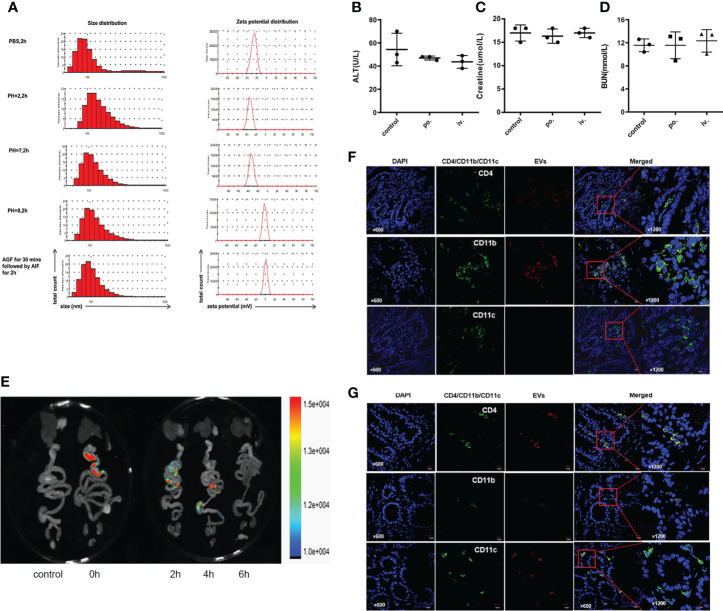
Stability and safety of EVs. **(A)** Size and zeta potential of EVs in different PH solutions and artificial gastrointestinal fluid. **(B–D)** Impact of EVs on liver and kidney function through oral and intravenous administration. Oral is abbreviated as po.; intravenous is abbreviate as iv.**(E)** Biodistribution of Dil-labeled EVs after gavage in the mouse. **(F, G)** Colocalization of Dil-labeled and CD4^+^T cells, CD11b^+^ follicular dendritic cells, and CD11c^+^macrophages in jejunum and ileum. Representative staining images of CD4, CD11b, CD11c (green), EVs (red) and DAPI(blue); scale bars, 10μm.

To test physicochemical stability, we incubated EVs in different PH environments and simulated gastrointestinal fluids for two hours (**Figure 2A**). The size of EVs slightly increased both in an acidic solution (PH=2) and in an alkaline solution (PH=8). EVs surface at neutral PH or in an acid environment was negatively charged, but weakly positive charged in an alkaline environment. Of note, incubation in gastric and intestinal enzymatic solution did not affect the heterogeneity of diameter and stability of the colloidal dispersion. In terms of drug safety, mice administrated with EVs preserved good hepatic or renal function, either orally or intravenously (**Figures 2B–D**).

To trace the biodistribution of EVs *in vivo*, we labeled EVs with the lipophilic dye Dil and performed FRI *ex vivo* scans of the gastrointestinal tract and other organs. Fluorescence accumulated predominantly in the ileocecum 2 to 4 hours after gavage (**Figure 2E**). Immunofluorescence colocalization results demonstrated that Dil-labeled EVs were co-stained with submucosa CD4^+^T cells, CD11b^+^ follicular dendritic cells, and CD11c^+^macrophages in the jejunum (**Figure 2F**) and ileum (**Figure 2G**).

### EVs-DexP inhibits lymphocytes activation *in vitro*

**Figure 3:**
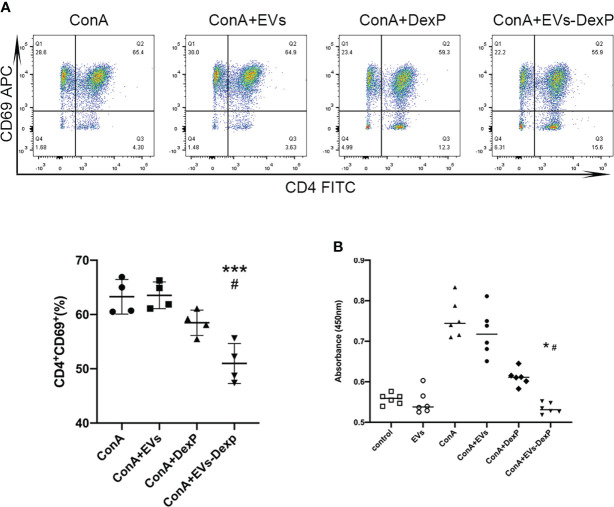
EVs-DexP inhibits lymphocytes activation *in vitro*. **(A)** Flow cytometry analysis of CD4^+^CD69^+^ population after the DexP or EVs-DexP treatment of PPs lymphocytes activated by ConA. From left to right, living cells, CD3^+^ cells, CD4^+^CD69^+^cells are detected. **(B)** CCK-8 assay showing the effect of EVs, DexP and EVs-DexP on PPs lymphocyte proliferation after co-culture for 24h. Data are presented as mean ± SD, * p<0.05 vs. ConA+EVs, *** p<0.001 vs. ConA+EVs, # p<0.05 vs. ConA+DexP; one-way ANOVA.

Flow cytometry revealed that DexP and EVs-DexP could considerably inhibit ConA stimulation on PPs lymphocytes(**Figure 3A**), although the difference between ConA+DexP and ConA or ConA+EVs was not statistically significant (ConA+DexP vs. ConA+EVs: g58.48 vs. 63.55, p=0.1265). EVs-DexP presented a more significant suppressing effect compared to DexP in that the proportion of CD4^+^CD69^+^ cells was dramatically reduced by EVs-DexP (ConA+DexP vs. ConA+EVs-DexP: 58.48% vs. 50.98%, p=0.0175). In addition, the CCK-8 experiment showed similar results (**Figure 3B**).

### Therapeutic efficacy of EVs-DexP in IgAN mice

**Figure 4:**
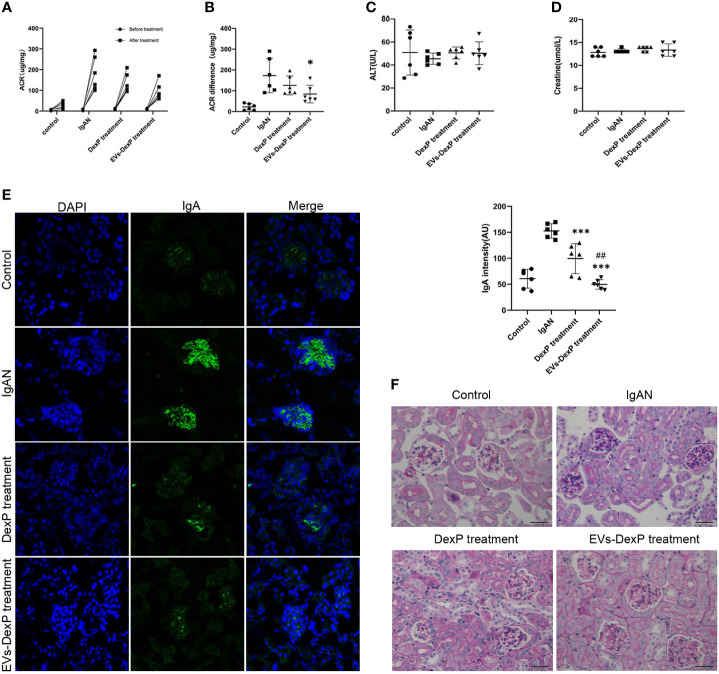
Improved therapeutic efficacy of EVs-DexP in IgAN mice. Twenty-four mice were randomized into four groups: control, IgAN model, IgAN receiving DexP treatment, and IgAN receiving EVs-DexP treatment. n=6 mice per group. **(A, B)** Spot-urine sample was collected before modeling and at the end of 12 weeks for urinary albumin/creatinine ratio(ACR) analysis. **(C, D)** Hepatic aminotransferase and serum creatine levels were measured at the end of 12 weeks. **(E)** Confocal images of immunofluorescence. IgA-FITC (green), DAPI was used to stain the nuclei(blue); scale bars, 75μm. **(F)** PAS staining images of glomerular; scale bars, 20μm. * p<0.05 vs. IgAN mice; ***p<0.001 vs. IgAN mice; ## p<0.01 vs. DexP treatment; one-way ANOVA.

In this study, we successfully constructed an IgAN mouse model using oral bovine serum albumin combined with intravenous staphylococcal enterotoxin B. The urinary albumin/creatinine ratio (ACR) was significantly higher after modeling (control vs. IgAN: 22.4 vs. 173.3 ug/mg, p<0.001)(**Figure 4A**). Light microscopy revealed mild to moderate mesangial cell proliferation, as well as immunofluorescence presenting granular deposition of IgA in the mesangial area, as shown in the second row of graphs in **Figure 4E**.

During IgAN modeling, DexP or EVs-DexP was given every other day from the beginning of the 6th week to the end of the 12th week. Both DexP and EVs-DexP treatment reduced urine protein to varied degrees, as shown in **Figure 4B** by the ACR difference before and after treatment (IgAN vs. DexP treatment: 173.3 vs.126.1 ug/mg, p=0.420; IgAN vs. EVs-DexP treatment: 173.3 vs. 84.3 ug/mg, p=0.037). EVs-DexP seemed to alleviate proteinuria to a more considerable extent than DexP despite no significant statistical difference in ACR (DexP treatment vs. EVs-DexP treatment: 126.1 vs. 84.3ug/mg, p=0.523) (**Figure 4B**). Hepatic aminotransferase (**Figure 4C**) and serum creatine (**Figure 4D**) fluctuated within normal limits in the course of modeling and treatment.

As seen in **Figure 4F**, the IgAN mice displayed diffuse mild to moderate mesangial cell proliferation with focal segmental hyperplasia and occasional sclerosis, graded as Lee’s grade III. DexP treatment resulted in less extent of mesangial proliferation and IgA intensity than IgAN counterparts, especially the IgAN mice given EVs-DexP only displayed mild segmental mesangial proliferation with faint IgA deposition, scoring Lee’s grade I-II.

### EVs-DexP decreased intestinal IgA^+^ lymphocytes

**Figure 5:**
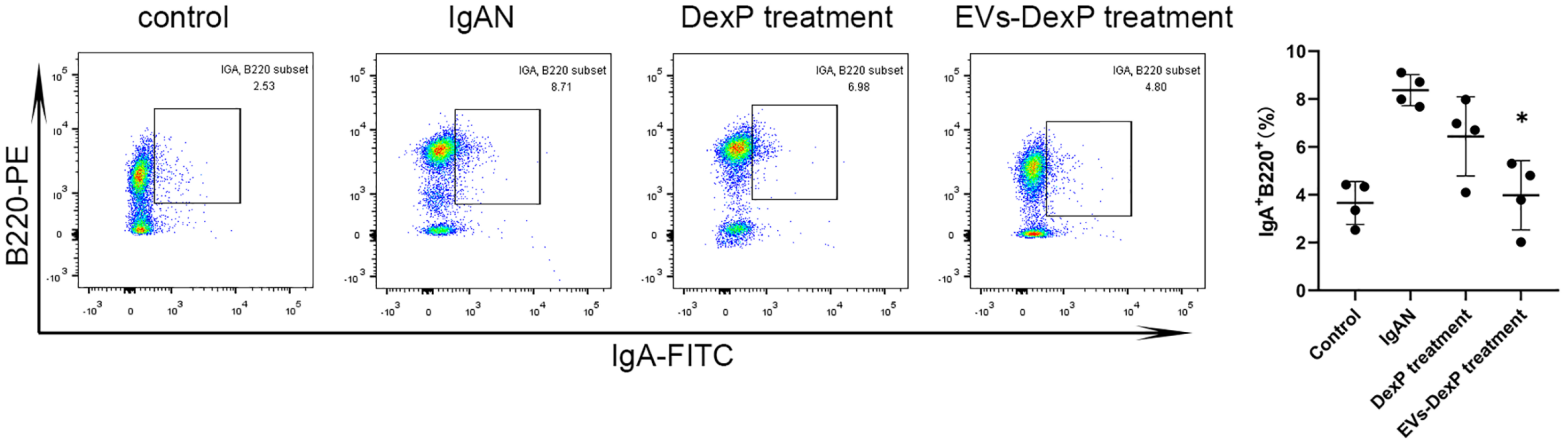
Effect of EVs-DexP on IgA^+^lymphocytes immunophenotype in PPs. Flow cytometry analysis of the ratio of IgA^+^ B220^+^ cells in PPs from different intervention groups of IgAN mice. n=4 mice per group.* p<0.05 vs. IgAN mice; one-way ANOVA.

IgA-secreting plasma cells have been discovered to derive from the antigen-specific IgA-committed B cells in PPs. IgA can be expressed in activated B lymphocytes, plasmablasts, and mature plasma cells. B220 is a surface antigen molecule primarily expressed on B lymphocytes (IgA^+^ B220^+^cells) and fades when B cells develop into mature plasma cell (IgA^+^B220^–^cells). We then examined the immunophenotype alterations of lymphocytes in PPs of each group. As seen in **Figure 5**, the ratio of IgA^+^ B220^+^ in PPs was much higher in the IgAN group, whereas DexP and EVs-DexP were able to drastically lower this ratio (IgAN vs. DexP treatment: 8.37% vs. 6.44%, p=0.275; IgAN vs. EVs-DexP treatment: 8.37% vs. 3.98%, p=0.016). However, the difference in the ratio of IgA^+^B220^+^ cells in PPs was marginal between the two treatment groups (DexP vs. EVs-DexP: 6.44% vs. 3.98%, p=0.216).

### EVs-DexP reduced intestinal IgA synthesis

**Figure 6:**
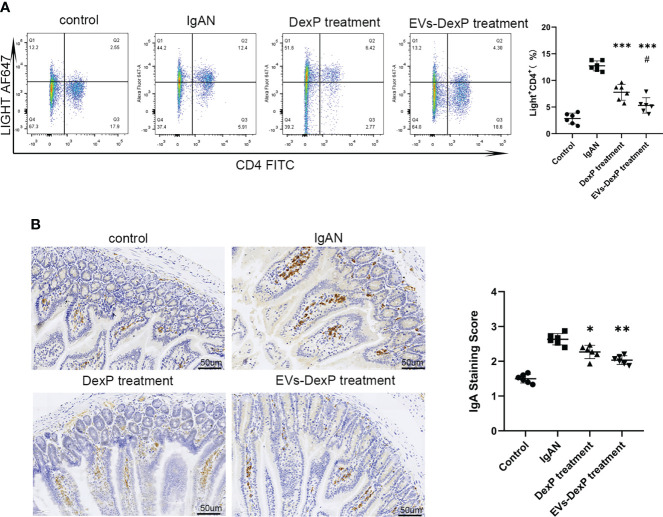
Effect of EVs-DexP on intestinal IgA production. **(A)** Flow cytometry analysis of the ratio of LIGHT^+^CD4^+^ cells in PPs from different intervention groups of IgAN mice. **(B)** Immunohistochemical staining intensity of IgA in the intestine; scale bars, 50μm. Data are presented as mean ± SD of six independent mice per group. *p<0.05 vs. IgAN mice, **p<0.01 vs. IgAN mice,*** p<0.001 vs. IgAN mice; # p<0.05 vs. DexP treatment; one-way ANOVA.

LIGHT is a costimulatory molecule expressed on activated T cells. In the PPs of IgAN mice, the percentage of LIGHT^+^CD4^+^ cells were decreased by both DexP and EVs-DexP (IgAN vs. DexP treatment: 21.05% vs. 11.97%, p<0.0001; IgAN vs. EVs-DexP treatment: 21.05% vs. 7.31%, p<0.0001). EVs-DexP exhibited stronger suppressive effect than DexP (DexP vs. EVs-DexP treatment: 11.97% vs. 7.31%, p=0.0318) (**Figure 6A**). Immunohistochemistry of the intestine (PPs removed for flow cytometry assay) revealed that IgA staining of the IgAN group was much stronger than the other two treatment groups, and EVs-DexP could significantly reduce IgA production (**Figure 6B**).

## Discussion

In this study, we successfully developed an extracellular vesicles-based delivery system encapsulated with dexamethasone(EVs-DexP), which displayed advantages over DexP in alleviating renal IgA deposition, reducing intestinal IgA production, accompanied by decreased ratio of IgA^+^B220^+^ lymphocytes in Peyer’s patches.

Clinical observations elicited interest in mocosal immunity in patients with IgAnephropathy that those who experience episodes of gross hematuria often have a history of upper respiratory or intestinal preceding infection. Inflammatory bowel diseases such as ulcerative colitis and Crohn’s disease are often combined with IgA nephropathy (11, 12).Besides, IgAN susceptibility loci can be divided into two categories according to the relationship with the intestinal disease (13, 14): the first category share with inflammatory disease (IBD)susceptibility loci such as HLA-DQ/DR, CARD9 and HORMAD2; the second involves genes encoding for the maintenance of intestinal immune barrier integrity and regulation of intestinal mucosal immune response such as DEFA, TNFSF13, VAV3, ITGAM-ITGAX and PSMB8. Based on a better knowledge of mucosal immunity, B cell activation, and complement activation in IgA nephropathy, several clinical studies of targeted treatments are currently underway. B cell–depleting therapy with rituximab, which was originally developed for the treatment of rheumatoid arthritis and B cell malignancies, is an appealing therapeutic option, especially because B cells may be involved in the production of galactose-deficient IgA1 and its antibodies in IgA nephropathy. Despite the fact that rituximab effectively reduced CD19^+^B cells, it did not improve eGFR or reduce proteinuria when compared to supportive therapy. Furthermore, neither the serum levels nor the antibodies of galactose-deficient IgA1 were lowered (15). Interestingly, rituximab had no beneficial benefits in patients with ulcerative colitis (16), comparable to the results in IgA nephropathy. More notably, a novel enteric capsule formulation of the locally acting glucocorticoid budesonide (Nefecon) has gained much interest in phase 2a (17) and 2b (18) trials, in light of its considerable effect on urine albumin excretion through inhibiting intestinal immune activity. It was made to release the active component in the distal part of the ileum and the proximal part of the colon, where the Peyer’s patches are located. In addition, this targeted release corticosteroids for IgA nephropathy were well tolerated when compared to systemic corticosteroids. The failure of rituximab and the clinical success of Nefecon in IgA nephropathy suggests a relationship between rituximab-resistant mucosal B cells and the continued production of IgA1, and supports the significance of mucosal immunity in IgA nephropathy.

Glucocorticoids have been a cornerstone of treatment for inflammatory and autoimmune renal disease. Because the available clinical evidence (2, 3) suggests a significant risk of toxicity associated with high-dose corticosteroid therapy in IgAN, the KDIGO guidelines state that “clinical benefit of corticosteroids in IgAN is not established and should be given with extreme caution or avoided entirely in the situations”. The challenge for improving IgAN treatment is to strike a balance between efficacy and risk of systemic steroid-based regimens. Dexamethasone (Dex) is one of the highly potent glucocorticoids applied to treat a broad spectrum of allergic, hematologic, and autoimmune diseases. Regarding its long half-life (36-54 hours) and significant adrenal suppression, Dex is rarely prescribed to treat glomerulonephritis compared to short-acting prednisone or methylprednisone. However, low dosage and local application have been proven to exert its anti-inflammation effect while alleviating adverse effects occurring in systemic administration, such as infection, osteoporosis, and hyperglycemia.

Extracellular vesicles are a highly heterogeneous class of vesicles released by all types of cells for the purpose of mediating intercellular communication. EVs are taken up by the target cells when utilized as drug delivery vehicles. Edible plant nanoparticles (19) were reported to be similar in sizes and structures to mammalian derived EVs, possessing a stable physicochemical property, biosafety, and large-scale production. Therefore, they have garnered growing interest as promising new candidates in the field. Ju et al. (20) published the first report that grape exosome-like nanoparticles can penetrate the intestinal mucus barrier, be taken up by mice intestinal stem cells. Wang et al. (21)illustrated that grapefruit-derived nanovesicles were preferentially taken up by intestinal macrophages and ameliorated DSS-induced mouse colitis. Oral administration provides lots of advantages over other administration methods, the most compelling of which is a reduction in systemic exposure. We found that orange-derived EVs maintained their stability in the gastrointestinal tract, which is crucial for the oral delivery approach applied to treat other diseases that require steroid minimization. Following gavage, orange-derived EVs accumulated in the ileocecum of mice, where they were picked up by dendritic cells and monocytes located in the submucosal PPs. This motivated us to design a targeted oral formulation for IgAN that acts on intestinal lymphocytes.

Therefore, we attempted to encapsulate orange-derived EVs with water-soluble DexP. *In vitro*, we confirmed that EVs-DexP had a more potent suppressive effect on stimulated lymphocytes than DexP. Notably, in the IgAN mouse model, as compared to the free DexP, EVs-DexP displayed advantages over DexP in reducing proteinuria and alleviating mesangial hyperplasia and IgA deposition, even though albuminuria following therapy was not statistically significant in both groups. Munagala and colleagues (22) identified bovine milk as a scalable source of exosomes capable of delivering chemotherapeutic agents. In their report, drug-loaded exosomes showed much greater effectiveness in cell culture experiments and against lung tumor xenografts *in vivo* when compared to a free drug. Subsequently, they demonstrated that milk-derived exosome loading with antitumor agents might serve as an alternative to conventional intravenous treatment to boost efficacy and minimize toxicity (23, 24). In accordance with the findings of earlier studies (25), we assume that exosomes improve the benefit/risk ratio of medications by enhancing their bioavailability, selectivity, and effectiveness in the target tissue while reducing the required doses and minimizing adverse effects to healthy tissues.

As the induction site of mucosal immunity, PPs are lymph nodes structured into a subepithelial dome rich in CD11c^+^ dendritic cells, T cell-rich interfollicular zones, and B cell-rich follicles with a high frequency of IgA^+^ cells. B lymphocytes undergo allotype conversion to IgA^+^ B lymphocytes in PPs when they are at rest. Unlike the spleen and other lymph nodes, IgA secreting B lymphocytes in PPs do not directly differentiate into mature plasma cells in PPs, but rather leave at plasma blast stage, lose their B lymphocytes label, enter into lymphocyte recycling, and eventually reach the lamina propria of the small intestine, where they develop further into mature plasma cells (26). Following EVs-DexP exposure, IgA^+^B220^+^ lymphocytes in PPs decreased, and the intestine secreted less IgA. As a consequence, we reasoned that EVs-DexP reduced proteinuria and IgA deposition was directly related to decreased intestinal IgA production by dampening the activation of IgA^+^ B cells in PPs.

The synthesis of IgA in mucosal areas is initiated by T lymphocyte-dependent B cell responses or T-cell independent mechanisms. The IgA produced in a T-dependent manner is like a general response establishing specific immunity induced by pathogens with protective high-affinity. The IgA produced by B-lymphocytes in a T-independent context is often against commensal bacteria sharing an interspecies reactivity. LIGHT (homologous to lymphotoxin, exhibits inducible expression, competes with herpesvirus glycoprotein D for HVEM on T cells), a member of the TNF superfamily (TNFSF14), is a proinflammatory cytokine and potent costimulatory molecule (27)expressed on activated T cells (28). Previous findings establish a crucial role for LIGHT in T cell activation *via* direct stimulation of neighboring T cells (27). Wang et al. demonstrate a direct contribution of T cell-mediated mucosal immunity in the development of IgAN (29). In their study, LIGHT transgenic mice acquired intestinal inflammation with dysregulated polymeric IgA production, transportation, and clearance caused by T cells. This model produced elevated levels of polymeric IgA, anti-DNA IgG, and IgA antibodies, as well as IgA and C3 mesangial deposition accompanied by proteinuria and hematuria, highlighting a direct role of T-cell-mediated mucosal immunity to IgAN pathogenesis. In mice with a human LIGHT transgene expressed under the control of the CD2 promoter (30), T lineage cells constitutively express the transgene-derived LIGHT, and as a result, the mice developed inflammation and tissue destruction. The inflammation in the intestine was particularly severe. This correlated with other changes in the mucosal immune system, such as selective increases in lamina propria B cells and increases in serum IgA, despite decreases in B lymphocytes elsewhere. The T cell-mediated terminal differentiation of plasma cells to IgA secretion are up-regulated during inflammation. Similar to the previous reports, our findings demonstrated that the IgAN animals had a higher ratio of LIGHT^+^CD4^+^ cells and IgA^+^B220^+^ cells, and that EVs-DexP repressed both, indicating that intestinal IgA production was reduced *via* a T-cell-dependent mechanism.

## Conclusions

In conclusion, our research revealed the stability, safety, and capacity to target intestine lymphocytes of orange-derived EVs as an oral drug delivery vehicle. Notably, EVs encapsulated with DexP displayed advantages over free DexP in inhibiting lymphocyte activation, reducing proteinuria, and alleviating renal pathological lesions in IgAN animals. Given their potential use in clinical practice, further investigation on dose-response, pharmacokinetics, and adverse effects analysis is warranted. In that oranges are a widely available and economically reliable source for EVs, and dexamethasone is a common and highly potent glucocorticoid, the EVs-DexP oral formulation is expected to serve as an alternative for steroid-based therapy, enhancing efficacy while minimizing systematic exposure. Our findings contribute to a better understanding of the importance of intestine immune in the development of IgAN.

## Data availability statement

The original contributions presented in the study are included in the article/**Supplementary Material**. Further inquiries can be directed to the corresponding author.

## Ethics statement

The animal study was reviewed and approved by the Institutional Animal Care and Use Committee (IACUC) of Nanfang Hospital.

## Author contributions

WZ and GW conceptualized and supervised the study. WZ and YY performed experiments and wrote the manuscript. XL and LY isolated EVs and performed characterization analysis and drug loading. JL and ZZ performed renal tissue sectioning, staining, and pathology scoring. All authors contributed to the article and approved the submitted version.

## Funding

The research was funded by the National Natural Science Foundation of China (No. 81870489).

## Conflict of interest

The authors declare that the research was conducted in the absence of any commercial or financial relationships that could be construed as a potential conflict of interest.

## Publisher’s note

All claims expressed in this article are solely those of the authors and do not necessarily represent those of their affiliated organizations, or those of the publisher, the editors and the reviewers. Any product that may be evaluated in this article, or claim that may be made by its manufacturer, is not guaranteed or endorsed by the publisher.

## Supplementary material

Supplementary Figure 1 Graphical abstract. We obtained a considerable amount of EVs from oranges as an oral drug delivery vehicle targeting intestinal lymphocytes. EVs encapsulated with DexP displayed advantages over free DexP in inhibiting lymphocyte activation, reducing proteinuria, and alleviating renal pathological lesions in IgAN animals. EVs-DexP oral formulation is likely to serve as an alternative for traditional steroid-based therapy for IgAN, improving efficacy while minimizing systematic exposure.

Supplementary Figure 2 FACS gating strategy of CD4^+^CD69^+^ population after the DexP or EVs-DexP treatment of PPs lymphocytes activated by ConA.

Supplementary Figure 3 FACS gating strategy of IgA^+^B220^+^ cells in PPs.

Supplementary Figure 4 FACS gating strategy of LIGHT^+^CD4^+^ cells in PPs.
