# Corrigendum: Orange-derived and dexamethasone-encapsulated extracellular vesicles reduced proteinuria and alleviated pathological lesions in IgA nephropathy by targeting intestinal lymphocytes

**DOI:** 10.3389/fimmu.2022.1047951

**Published:** 2022-10-12

**Authors:** Wang Zhang, Ye Yuan, Xiang Li, Jiao Luo, Zhanmei Zhou, Lei Yu, Guobao Wang

**Affiliations:** ^1^ Renal Division, Nanfang Hospital, Southern Medical University, National Clinical Research Center for Kidney Disease, State Key Laboratory of Organ Failure Research, Guangzhou, China; ^2^ Department of Anatomy, School of Basic Medical Sciences, Southern Medical University, Guangzhou, China

**Keywords:** IgA nephropathy, intestine immunity, extracellular vesicle (EV), immunosuppressive therapy, Peyer’s patches

In the published article, there was an error in the Funding statement The grant number was incorrect. The correct Funding statement appears below:

“The research was funded by the National Natural Science Foundation of China (No. 81870489)”.

The authors apologize for this error and state that this does not change the scientific conclusions of the article in any way. The original article has been updated.

## Publisher’s note

All claims expressed in this article are solely those of the authors and do not necessarily represent those of their affiliated organizations, or those of the publisher, the editors and the reviewers. Any product that may be evaluated in this article, or claim that may be made by its manufacturer, is not guaranteed or endorsed by the publisher.

