# Supplementary material for: Orange-derived and dexamethasone-encapsulated extracellular vesicles reduced proteinuria and alleviated pathological lesions in IgA nephropathy by targeting intestinal lymphocytes
Source: Front Immunol. 2022 Aug 31;13:900963. doi: 10.3389/fimmu.2022.900963 (PMC9471245; doi:10.3389/fimmu.2022.900963)
Supplement: Supplementary file 1 [file Table_1.docx]

Table 1. The ratio of CD4^+^CD69^+^ cells in vitro by flow cytometry analysis.

|  |  | Mean1 | Mean2 | P |
| --- | --- | --- | --- | --- |
| ConA+DexP vs ConA | | 58.48±2.03 | 63.28±2.76 | 0.1560 |
| ConA+DexP vs ConA+EVs | | 58.48±2.03 | 63.55±2.13 | 0.1265 |
| ConA+DexP vs ConA+EVs-DexP  ConA+EVs vs ConA+EVs-DexP | | 58.48±2.03  63.55±2.13 | 50.98±3.20  50.98±3.20 | 0.0175  0.0003 |

Table 2. The urinary albumin/creatinine ratio of IgAN mice at the end of 12 weeks.

|  |  | Mean1 | Mean2 | P |
| --- | --- | --- | --- | --- |
| DexP vs Control | | 126.08±41.61 | 22.44±14.33 | 0.0129 |
| DexP vs IgAN | | 126.08±41.61 | 173.29±75.31 | 0.4204 |
| DexP vs EVs-DexP  IgAN vs EVs-DexP | | 126.08±41.61  173.29±75.31 | 84.31±38.76  84.31±38.76 | 0.5233  0.0367 |

Table 3.The ratio of IgA^+^ B220^+^ cells in PPs of IgAN mice by flow cytometry analysis.

|  |  | Mean1 | Mean2 | P |
| --- | --- | --- | --- | --- |
| DexP vs Control | | 6.44±1.65 | 3.66±0.90 | 0.1142 |
| DexP vs IgAN | | 6.44±1.65 | 8.37±0.65 | 0.2753 |
| DexP vs EVs-DexP  IgAN vs EVs-DexP | | 6.44±1.65  8.37±0.65 | 3.98±1.45  3.98±1.45 | 0.2162  0.0160 |

Table 4. The ratio of LIGHT^+^CD4^+^ cells in PPs of IgAN mice by flow cytometry analysis.

|  |  | Mean1 | Mean2 | P |
| --- | --- | --- | --- | --- |
| DexP vs Control | | 11.97±0.82 | 8.92±1.35 | 0.238 |
| DexP vs IgAN | | 11.97±0.82 | 21.05±4.08 | <0.0001 |
| DexP vs EVs-DexP  IgAN vs EVs-DexP | | 11.97±0.82  21.05±4.08 | 7.31±2.18  7.31±2.18 | 0.0318  <0.0001 |
