# Supplementary figures and images for: Orange-derived and dexamethasone-encapsulated extracellular vesicles reduced proteinuria and alleviated pathological lesions in IgA nephropathy by targeting intestinal lymphocytes
Source: Front Immunol. 2022 Aug 31;13:900963. doi: 10.3389/fimmu.2022.900963 (PMC9471245; doi:10.3389/fimmu.2022.900963)

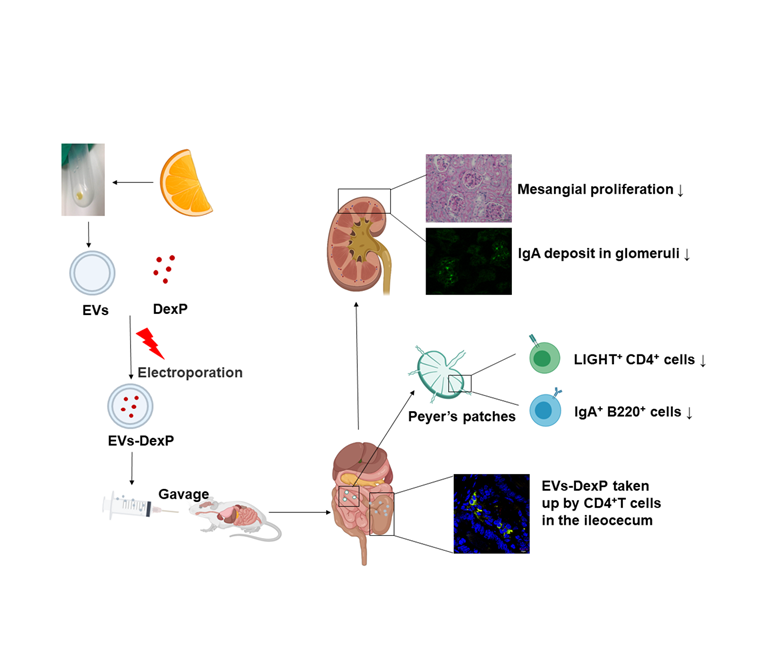

Supplement: Supplementary Figure 1 — Graphical abstract. We obtained a considerable amount of EVs from oranges as an oral drug delivery vehicle targeting intestinal lymphocytes. EVs encapsulated with DexP displayed advantages over free DexP in inhibiting lymphocyte activation, reducing proteinuria, and alleviating renal pathological lesions in IgAN animals. EVs-DexP oral formulation is likely to serve as an alternative for traditional steroid-based therapy for IgAN, improving efficacy while minimizing systematic exposure. [file Image_1.tif]

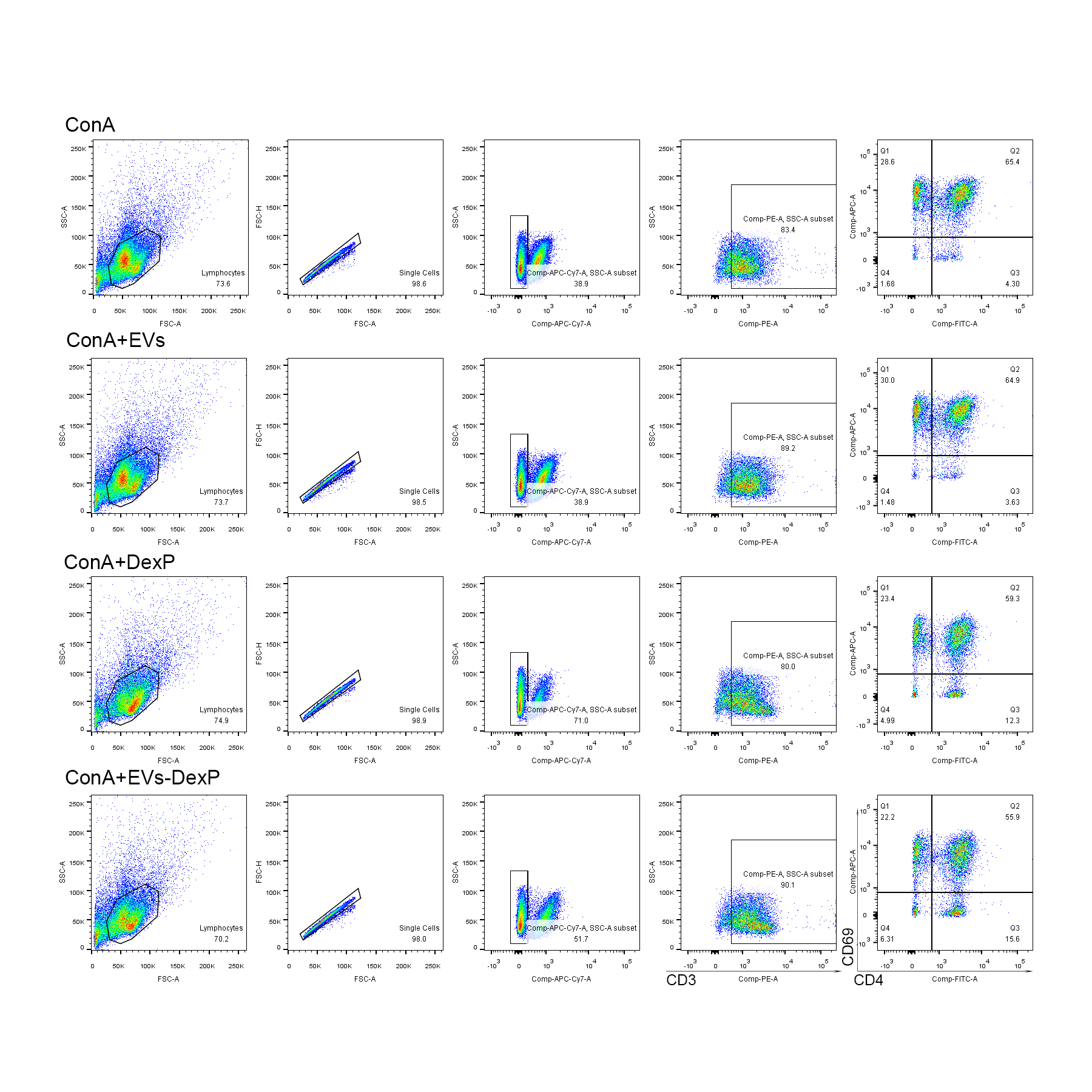

Supplement: Supplementary Figure 2 — FACS gating strategy of CD4+CD69+ population after the DexP or EVs-DexP treatment of PPs lymphocytes activated by ConA. [file Image_2.tif]

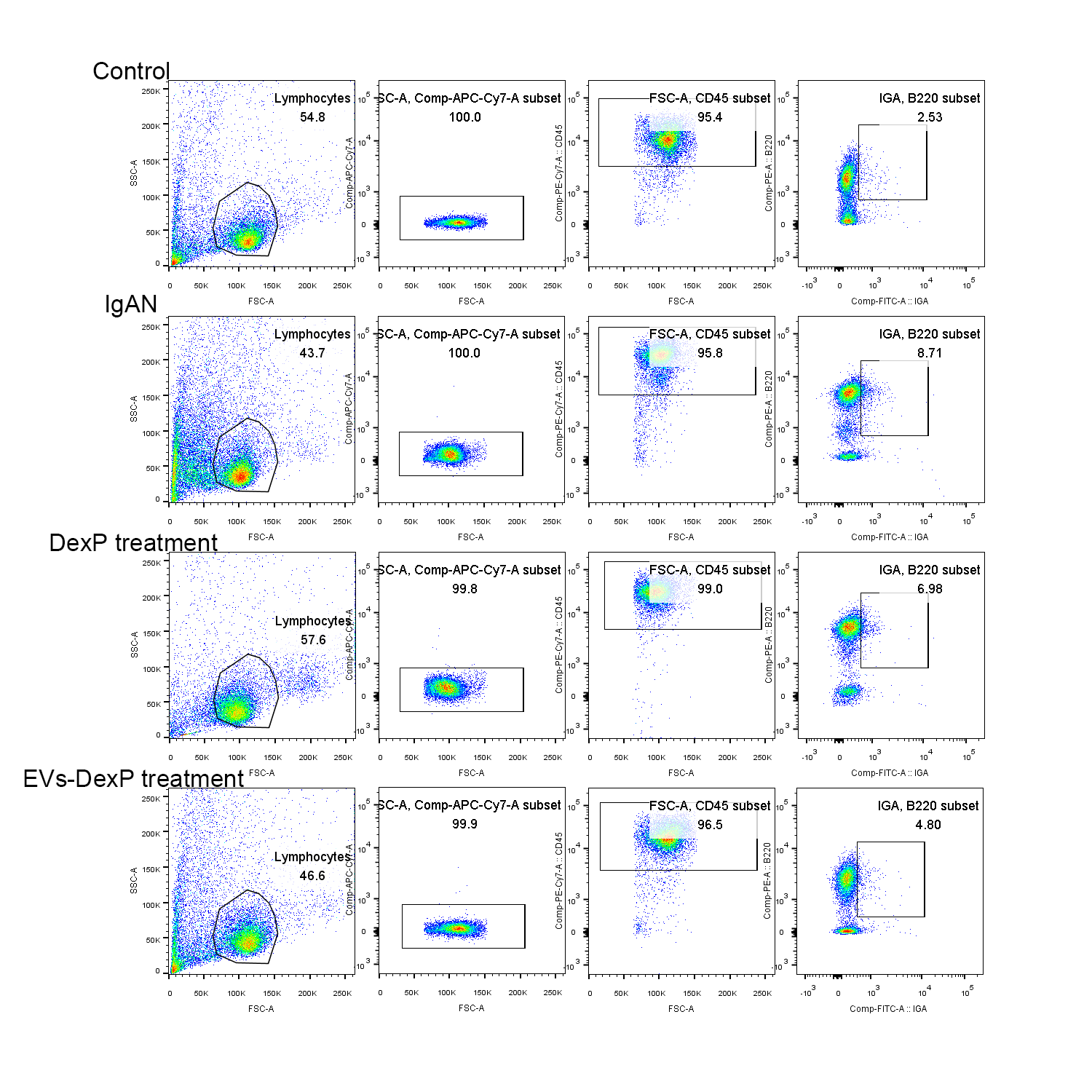

Supplement: Supplementary Figure 3 — FACS gating strategy of IgA+B220+ cells in PPs. [file Image_3.tif]

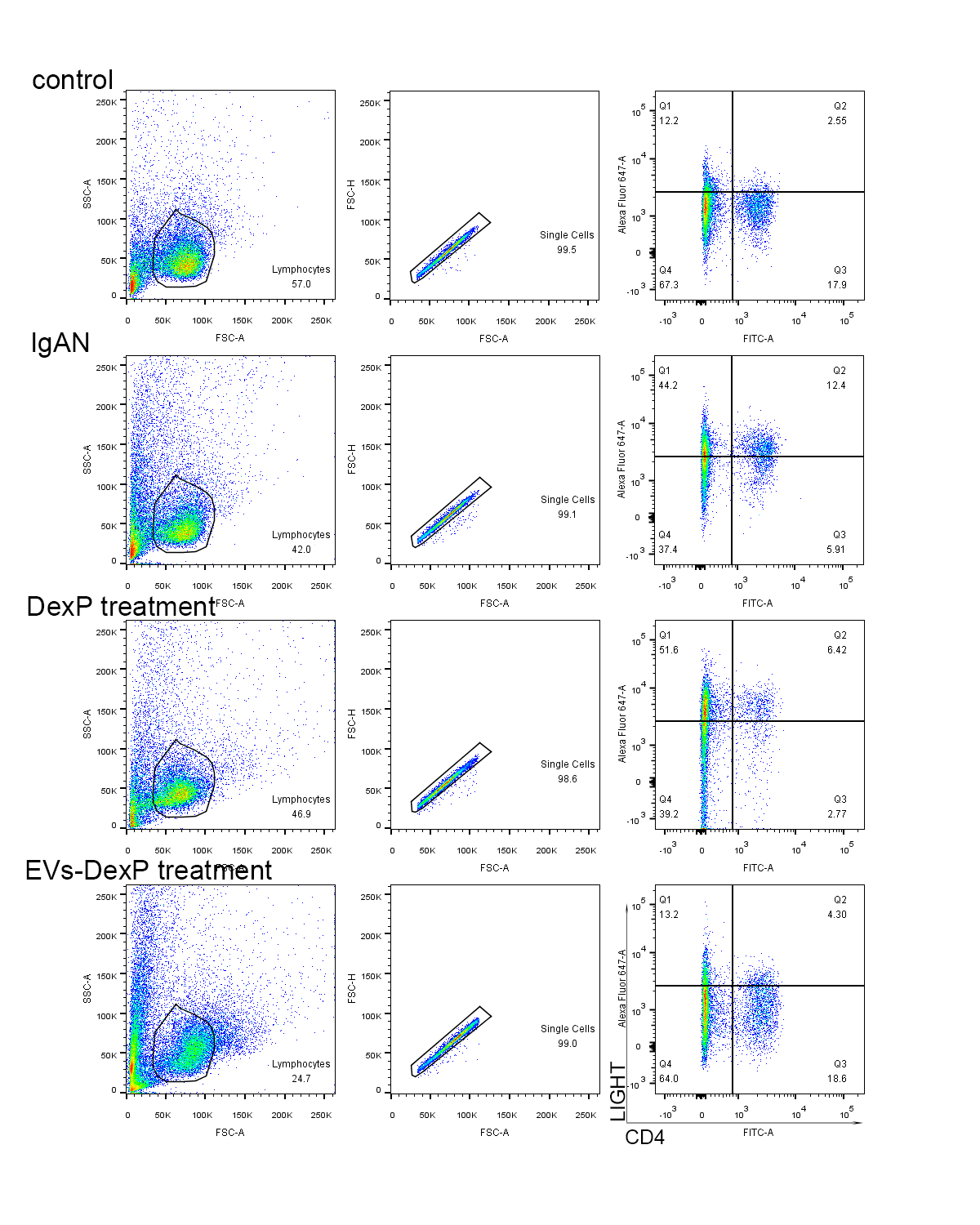

Supplement: Supplementary Figure 4 — FACS gating strategy of LIGHT+CD4+ cells in PPs. [file Image_4.tif]
